# COVID-19 infection and vaccines: potential triggers of Herpesviridae reactivation^☆^

**DOI:** 10.1016/j.abd.2022.09.004

**Published:** 2023-02-10

**Authors:** Alba Navarro-Bielsa, Tamara Gracia-Cazaña, Beatriz Aldea-Manrique, Isabel Abadías-Granado, Adrián Ballano, Isabel Bernad, Yolanda Gilaberte

**Affiliations:** Department of Dermatology, Miguel Servet University Hospital, IIS Aragón, Zaragoza, Spain

**Keywords:** Coinfection, COVID-19, Herpesvirus 1, human, Herpesvirus 2, human, Latent infection

## Abstract

Since the onset of the COVID-19 outbreak, numerous articles have highlighted a possible link between COVID-19 vaccination or infection and Herpesviridae co-infection or reactivation. The authors conducted an exhaustive literature review on this topic, the results of which are presented individually for each member of the Herpesviridae family: Herpes Simplex Virus (HSV) types-1 (HSV-1) and 2 (HSV-2); Varicella-Zoster Virus (VZV); Epstein-Barr Virus (EBV); Cytomegalovirus (CMV); HHV-6; HHV-7; and HHV-8. These human herpesviruses can serve as prognostic markers for the COVID-19 infection and may even underlie some of the clinical manifestations initially attributed to SARS-CoV-2. In addition to SARS-CoV-2 infection, all corresponding vaccines approved to date in Europe appear capable of inducing herpesvirus reactivation. It is important to consider all viruses of the Herpesviridae family when managing patients infected with or recently vaccinated against COVID-19.

## Introduction

Human Herpesviruses (HHVs), which include Herpes Simplex Virus (HSV) types-1 (HSV-1) and 2 (HSV-2), Varicella-Zoster Virus (VZV), Epstein-Barr Virus (EBV), Cytomegalovirus (CMV), HHV-6, HHV-7, and HHV-8, are part of a family of DNA viruses that cause several diseases in humans. Studies of the relationship between these viruses and the recently emerged novel coronaviruses (SARS-CoV-2) have proposed the possibility that SARS-CoV-2 or SARS-CoV-2 vaccines may induce reactivation of different subtypes of these human herpesviruses.

In this review, the authors examine the available literature and provide an overview of reported cases of reactivation of different HHVs following COVID-19 infection or vaccination and consider the possibility that many manifestations originally attributed to COVID-19 may in fact be due to the reactivation of these viruses.

## Methods

The authors conducted a literature search (December 2021) of PubMed using the following terms, without restrictions: “COVID-19”, “SARS-CoV-2”, “COVID-19 Vaccines”, “human herpesviruses”, “human herpes virus”, “varicella-zoster virus”, “Epstein-Barr virus”, “cytomegalovirus”, “human herpes virus 1”, “human herpes virus 2”, “human herpes virus 6”, “human herpes virus 7”, and “human herpes virus 8”. Titles and abstracts of all types of studies, from case reports to reviews, were reviewed for relevance. Articles in English or Spanish were accepted. In cases of articles of uncertain relevance, the entire article was reviewed to determine its suitability for inclusion.

## Results

### Herpes simplex virus 1

An estimated 66% of the world’s population is infected with HSV-1. HSV-1 is mainly transmitted from person to person via infected oral secretions during close contact.^1^ After the initial infection, chronic infection is established in the sensory ganglia and reactivated on mucosa and skin. Although infections are frequently asymptomatic, they can produce a range of signs and symptoms. These include recurrent perioral or oral lesions (“cold sores”) and skin and mucous lesions.^2^

During HSV recurrences the reactivated virus switches from latent to lytic replication in trigeminal or sacral ganglia neurons and travels via anterograde axonal transport to epithelial cells, where it replicates and can be shed asymptomatically or give rise to clinically apparent ulceration.

The frequency of reactivation is increased in immunocompromised hosts, as containment of HSV requires intact cellular immunity. However, even in individuals with a vigorous humoral and cellular immune response, HSV-1 evades eradication via multiple immune evasion mechanisms. Human studies have demonstrated the key role of T-cells in immune surveillance and initial containment of HSV-2 reactivation in the mucosa and in ganglia.^3^ Immune compromise can be triggered by any one of a wide range of mechanisms, including infections by other viruses, such as SARS-CoV-2, which causes lymphopenia.^4^

Several articles have described HSV-1 reactivation in patients with COVID-19. Le Balch et al.^5^ published the first series of 36 cases of HSV-1 reactivation in critically ill COVID patients, and reported higher rates than those described in earlier studies of critically ill patients. Since then, many such cases have been reported, ranging from mild (e.g., HSV lesions on the lip) to more serious manifestations, including two fatal cases of acute liver failure secondary to HSV-1 and cranial polyneuropathy, respectively.

HSV-1 has been detected in many different tissues in COVID-19 patients. In May 2020 Hu et al.^6^ demonstrated the presence of latent HSV-1 in the human anterior segment and the aqueous humor of SARS-CoV-2-infected patients, and speculated that latent HSV-1 infection may have been activated in response to immune compromise caused by COVID-19 infection. This hypothesis was subsequently confirmed in multiple cases of HSV-1 keratitis.^7^

While several cutaneous and mucosal manifestations were initially directly attributed to SARS-CoV-2, it was later suggested that some, including oral lesions, could be the result of the reactivation of other, more common viruses. Kämmerer et al.^8^ detected anti-HSV IgM in serum samples from a patient who developed herpetic gingivostomatitis, and Kano et al.^9^ reported a case of herpetic glossitis related to COVID-19 treatment.

The coexistence of HSV-1 and SARS-CoV-2 is also considered in the clinical management of COVID-19 patients: pulmonary HSV-1 reactivation in invasively ventilated patients is a known phenomenon, and tracheal secretions and bronchial lavage samples are screened by Polymerase Chain Reaction (PCR) for HSV-1. This reactivation of HSV-1 coincides with a decrease in the expression of interferon-stimulated genes and an increase in highly activated CD38+HLADR+ CD8 T-cells in the later stages of SARS-CoV-2-infection.^10^

With the development of new vaccines for SARS-CoV-2, reports of HSV-1 reactivation in recently inoculated individuals began to appear. This correlation was observed in the UK’s yellow card scheme for recording adverse incidents with medicines, and a total of 369 cases of HSV infections associated with COVID-19 vaccines are recorded in the European database of suspected adverse drug reactions (EudraVigilance) (Table 1): 57 after CX-024414; 305 after Tozinameran; 118 after CHADOX1 NCOV-19; and 7 after AD26.COV2.S.^11^ Reporting bias is very likely, given that HSV-1 reactivations are likely underdiagnosed due to their innocuous nature.

**Table 1:** Cases of herpesvirus infections associated with COVID-19 vaccines are recorded in the European database of suspected adverse drug reactions (EudraVigilance).

Virus	COVID-19 MRNA vaccine moderna (CX-024414)	COVID-19 MRNA vaccine Pfizer-Biontech (Tozinameran)	COVID-19 vaccine Astrazeneca (CHADOX1 NCOV-19)	COVID-19 vaccine Janssen (AD26.COV2.S)	Total
HSV-1	57	305	118	7	369
HSV-2	41	200	116	8	365
VZV	1410	9652	3054	205	14321
EBV	12	38	12	1	63
CMV	10	39	6	1	56
HHV-6	1	9652	1	0	9654
HHV-7	0	0	0	0	0
KSHV	0	3	0	0	3
Total	1531	19889	3307	222	24831

HSV-1, Herpes Simplex Virus type-1; HSV-2, Herpes Simplex Virus type-2; VZV, Varicella-Zoster Virus; EBV, Epstein-Barr Virus; CMV, Cytomegalovirus; HHV-6, Herpes Virus-6; HHV-7, Herpes Virus-7; KSHV, Kaposi Sarcoma-associated Herpesvirus.

### Herpes simplex virus 2

HSV-2 is a ubiquitous pathogen that primarily causes genital infections. Genital herpes is one of the most common sexually transmitted infections worldwide. Although HSV-2 remains the main cause of recurrent genital herpes infections (70%–90% overall), the proportion due to HSV-1 has been increasing. Primary, non-primary initial, and recurrent infections can be symptomatic or asymptomatic. Notably, 81% of those with positive serology have no prior diagnosis of genital HSV infection. Genital HSV-2 infection also increases the risk of acquiring and transmitting HIV infection.^12^ PCR is increasingly used as a more rapid, sensitive, and specific method to detect HSV DNA in samples from the skin and other organs.

To date, there is only 1 reported case of SARS-CoV-2 infection and concomitant infection with human immunodeficiency virus, HSV-2, and SARS-CoV-2, with a fulminant clinical course and a false-negative real-time PCR result.

A total of 365 cases of HSV-2 following vaccination against COVID-19 are recorded in the EUDRAvigilance database (Table 1): 41 after CX-024414; 200 after Tozinameran; 116 after CHADOX1 NCOV-19; and 8 after AD26.COV2.S.^11^

### Varicella-zoster virus

Herpes Zoster (HZ) is associated with high morbidity. It is caused by the reactivation of latent HZV following a decline in cell-mediated immunity, resulting in the appearance of clustered metamerically distributed papules on an erythematous base that rapidly evolves into vesicles, which in turn can progress to pustules. HZ has been associated with advanced age but can occur in individuals with diseases and those treated with immunosuppressant drugs and can even affect immunocompetent individuals. The authors now know that SARS-CoV-2 infection affects T-lymphocytes, especially CD4+T cells, CD8+T cells, and natural killer cells, resulting in an immunocompromised state that may trigger the reactivation of latent viral infections.^13^ This association first came to light when increases in the number of HZ cases were observed during the COVID-19 outbreak relative to the corresponding period in 2019 in several different countries, including Turkey and Brazil.^14^

Case reports and series have described HZ following COVID-19, occurring most frequently within 1–2 weeks of COVID-19 infection, in the majority of cases with a typical presentation affecting a single dermatome. Atypical HZ presentations have been reported, especially in patients with lymphopenia, giving rise to multimetameric involvement and skin necrosis, and, in the most severe cases, central retinal vein occlusion, meningitis, and encephalomyelitis.^15^

Elderly immunocompromised patients are more susceptible to severe and fatal HZ. HZ has been proposed as a clinical biomarker of COVID-19: it has been postulated that ophthalmic involvement may constitute a complication or indicator of COVID-19 infection, particularly in young, immunocompetent patients and in pregnant women.^16^

This association has been investigated by bioinformatic analyses examining potential genetic crosstalk between HZ and COVID-19, and increases in Th17 cell differentiation and, consequently, IL-17 signaling have been described in both COVID-19 and HZ, suggesting that IL-17 signaling may be one mechanism through which COVID-19 can increase the risk of HZ.^17^

It is very likely that the prevalence of coinfection is underestimated, as many cases have been diagnosed by telemedicine, and the requisite laboratory testing for HZ was available only in a limited number of cases.^18^

There is more debate regarding COVID-19 with varicella (more commonly known as chickenpox) coinfection. Recalcati et al.^19^ first reported that varicella-like vesicles could constitute a cutaneous manifestation of COVID-19. Subsequently, Marzano et al.^20^ reported that 22 patients with a positive COVID-19 nasopharyngeal test developed varicella-like papulovesicular exanthem. This type of rash was related to typical clinical lesions and symptoms of herpes simplex/zoster. Llamas-Velasco et al.^4^ performed herpesvirus microarray PCR in 3 patients with SARS-CoV-2 and vesicular eruption, and detected VZV and other herpesvirus DNA sequences in the vesicle contents.

There are few cases of chickenpox in the context of a COVID-19 infection in which positive IgM antibodies against VZV have been detected or molecular analysis of the vesicle content has been performed.^21^

Generally, cases have been described in young people with clinical signs typical of chickenpox and SARS-CoV-2 infection, including high fever, myalgia, nasal discharge, and a rash consisting of vesicles, erosions, and crusts associated with petechial enanthem of the palatal mucosa.^21^ However, cases of more serious involvement with the development of varicella pneumonia have also been reported.^22^ This presumptive diagnosis must be established early to begin treatment with acyclovir, which prevents the progression of lung involvement, decreasing the likelihood of a fatal outcome.

Apart from laboratory tests to distinguish varicella-like exanthema from that caused by chickenpox, different pathological anatomical patterns have been described. In varicella-like exanthema, biopsy shows acantholysis and dyskeratosis with a suprabasal, unilocular intraepidermal vesicle, without the large multinucleated cells and ballooning degeneration typical of chickenpox.^23^ However, the incidence of varicella and other viral infections has dramatically decreased since the implementation of COVID-19 control measures.

Cases of HZ following the administration of SARS-CoV-2 vaccines have been reported worldwide. The largest series and narrative review include 399 cases, and another systematic review describes 91 cases of VZV reactivation following COVID-19 vaccination,^24^ counts lower than those who are registered in EudraVigilance (Table 1). The number of HZ cases associated with COVID-19 vaccines registered in the EudraVigilance database is 1410 for CX-024414, 9652 for Tozinameran, 3054 for CHADOX1 NCOV-19, and 205 for AD26.COV2.S.^11^

A systematic review by Katsikas Triantafyllidis et al.^24^ reported that the dermatomal distribution of skin lesions was variable among patients with HZ reactivation following COVID-19 vaccination, although the mammary region was the most frequently affected anatomical site. Most of the lesions and symptoms appeared after the administration of the first vaccine dose. There was a tendency towards greater susceptibility among patients aged > 60 years. Most patients were treated with oral valacyclovir in monotherapy. Furthermore, the majority of affected patients had a concomitant autoimmune disorder and/or were receiving immunosuppressant therapy.^24^

While causality has not yet been established, increased awareness and early recognition of HZ are crucial to ensure the optimal management of these patients. One of the most recent studies of the clinical and pathologic correlations of cutaneous COVID-19 vaccine reactions collects 4 cases of postvaccine HZ in which biopsies revealed viral cytopathic changes, leading the authors to propose that the immune response induced by vaccination may trigger reactivation of other viruses.

### Epstein Barr virus

EBV, human gammaherpesvirus 4, infects B-lymphocytes and epithelial cells. After primary infection EBV persists in a latent state in individual B-cells. Latent EBV is found in approximately 95% of the human population. The presence of EBV in B-lymphocytes has been associated with the appearance of lymphoproliferative diseases and autoimmune diseases such as multiple sclerosis, systemic lupus erythematosus, rheumatoid arthritis, and dermatomyositis.

Multiple factors have been implicated in EBV reactivation, in particular psychological stress, including the stress associated with hospitalization for several days. Reactivation is more likely to occur in immunosuppressed patients (e.g., transplant recipients).^25^ Several studies have described increased EBV reactivation in COVID-19 patients, especially those that are critically ill.^26^, ^27^ Paolucci et al.^26^ detected EBV DNA in 95.2% of COVID-19 patients hospitalized in an intensive care unit, as well as significant reductions in CD8+ cells and NK counts. This reactivation occurred earlier after admission to an Intensive Care Unit (ICU) and was associated with long-term hospitalization. The course of COVID-19 disease may be aggravated in patients that undergo EBV reactivation: these patients had a higher mortality rate and received more immuno-supportive treatment than SARV-CoV-2 patients without EBV coinfection.^28^ In fact, Solomay TV et al.^29^ reported that severe disease and pneumonitis were more common among patients with EBV coinfection. These patients have a more frequent fever, elevated inflammation markers (C-reactive protein), and increased liver enzyme levels.^30^ While mild alterations in liver function tests are common in patients with COVID-19 infection, marked elevation of liver enzymes should raise suspicion of EBV coinfection. These observations highlight the importance of distinguishing between EBV manifestations and COVID-19-associated complications.

One study reported EBV reactivations in 60% of patients with prolonged COVID-19 symptoms after resolution of acute disease, compared with 10% in a control group, suggesting that some long-term COVID-19 symptoms may be due to EBV reactivation.

Moreover, Verma et al.^31^ reported that EBV reactivation in nasopharyngeal and oropharyngeal epithelial cells increases expression of Angiotensin-Converting Enzyme 2 (ACE2), the cellular receptor for SARS-CoV-2, thereby favoring SARS-CoV-2 infection.^32^

The EUDRAvigilance database contains 63 cases of EBV reactivation after COVID-19 vaccination (Table 1): 12 for CX-024414; 38 for Tozinameran; 12 for CHADOX1 NCOV-19; and 1 for AD26.COV2.S.^11^ A single case of EBV-positive diffuse large B-cell lymphoma after the COVID-19 vaccine has been reported in a heart transplant recipient. One study reported no increase in herpesvirus reactivation, including EBV, 1-week after versus 1 week before COVID-19 vaccination (Tozinameran).^33^

### Cytomegalovirus

CMV is a member of the Herpesviridae family, and its prevalence increases with age. It typically causes primoinfection in young individuals and subsequently persists in a latent state throughout the life of the host.

In immunocompetent individuals, it remains asymptomatic. However, the increased rate of CMV reactivation among immunosuppressed patients, including solid organ transplant recipients and patients with AIDS or cancer, is a major concern and may require antiviral prophylaxis. This phenomenon has also been described in previously healthy patients admitted to an ICU, who are also at increased risk of reactivation of other herpesviruses.^34^ In fact, these rates appear even higher in critically ill COVID-19 patients, with viral reactivation reported in almost 50% of patients in the series by Le Balc'h et al.^5^ and other authors.^27^ Explaining this effect, at least in part, both severe lymphopenia and mechanical ventilation are known risk factors for secondary viral infections.

Many cases of CMV reactivation in COVID-19 patients have been reported, some with fatal consequences.^35^ CMV reactivation can manifest with a wide range of symptoms, including gastrointestinal bleeding and colitis, persistent fever, and secondary pneumonia, all of which are associated with a worse prognosis.^36^ Marked and persistent lymphopenia is characteristic of severe COVID-19 infection. Moreover, potent immunosuppressive agents, including high-dose corticosteroids and IL-6 antagonists such as tocilizumab, are more frequently used in these patients. These two factors may contribute to an increased risk of CMV reactivation in critically ill COVID-19 patients.^37^

Studies have investigated the role of CMV in regulating the immune response. CMV appears to contribute to accelerated immune senescence and exacerbate the cytokine storm associated with severe COVID-19 infection, thus negatively altering the host response to COVID-19 infection.^38^ Consequently, patients co-infected with COVID-19 and CMV appear to have a worse prognosis,^39^ and this immune response dysregulation may also explain why elderly, usually CMV-seropositive, patients develop more severe disease. In line with these findings, areas with a higher prevalence of CMV seropositivity also show higher COVID-19 mortality rates. A total of 56 cases of CMV reactivation after COVID-19 vaccination (Table 1) have been reported in Europe: 10 after CX-024414; 39 after Tozinameran; 6 after CHADOX1 NCOV-19; and 1 after AD26.COV2.S.^11^

### Human herpesvirus 6 and 7

Human Herpesvirus 6 (HHV-6) is a widespread beta-herpesvirus that is genetically related to human CMV. HHV-6 exhibits broad cell tropism in vivo and, like other herpesviruses, induces lifelong latent infection in humans. Many active HHV-6 infections, corresponding to primary infections, reactivations, or exogenous reinfections, are asymptomatic. However, the virus can cause serious diseases, particularly in immunocompromised individuals.

Cutaneous diseases associated with HHV-6 infection include roseola infantum, Pityriasis Rosea (PR), Gianotti-Crosti syndrome, drug reaction with eosinophilia and systemic symptoms, and thrombocytopenic purpura, among others.^40^ In the case of PR, several studies have established a causal role of systemic active HHV-6 and/or HHV-7 reactivation, based on the detection of viral DNA in the plasma of PR patients, the expression of mRNA and specific viral antigens, and detection by electron microscopy of HHV virions in PR lesions.^41^ Nonetheless, it is difficult to establish an etiological association between HHV-6 infection and cutaneous disease due to the ubiquity and near-universal prevalence of the virus and the limitations of current research tools.^40^

The association between SARS-CoV-2 and HHV-6 is supported by several reports during the COVID-19 pandemic describing an increased incidence of PR, and in one case HHV-6 reactivation was confirmed by serum PCR. The studied group detected positive IgM serology and/or IgG seroconversion for HHV-6 in 12 patients fulfilling COVID-19 clinical and/or microbiological criteria that presented with different cutaneous manifestations, including PR-like eruption.^42^ A case of a PR-like eruption in a COVID-19 patient with SARS-CoV-2 spike protein positivity on skin biopsy has also been reported, suggesting a direct pathogenic relationship with this virus, although the HHV-6 status of this patient was not investigated.

This reactivation has not only been described in association with PR but also in critically ill patients with COVID-19. In their series of 34 COVID-19 patients Simonnet et al.^27^ detected HHV-6 viremia in 7 (22%) patients, although HHV-6 status was not associated with morbidity and mortality. This percentage is close to the frequency reported in large studies of septic shock patients. One limitation of Simonnet et al.^27^ the study was absent a comparator population, which prevented the authors from determining whether SARS-CoV-2 infection or “ICU-acquired immunosuppression” were the main drivers of reactivation.

One report has described HHV-6 reactivation in the central nervous system of a COVID-19 patient with HHV-6 myelitis and concomitant myelin oligodendrocyte glycoprotein antibody-mediated parainfectious myelitis. Another reported positive detection of SARS-CoV-2, HSV-1, and HHV-6 nucleic acid in tear and conjunctival secretions from a non-conjunctivitis COVID-19 patient with obstruction of common lacrimal ducts.^6^ In both cases, it is speculated that latent HHV-6 infection may have been reactivated in the context of COVID-19-induced immune dysfunction.^6^

Recently, Biswas et al.^42^ reported a case of a child with the multisystem inflammatory syndrome (MIS) as a late complication of COVID-19. Initially, a high HHV-6 viral load was detected. Subsequently, the patient was found to have inherited chromosomally integrated HHV-6 (iciHHV-6). iciHHV-6 is HHV-6 that has been integrated into chromosomal telomeric regions and is transmitted through the germ line. This occurs in ∼1% of individuals. The presence of iciHHV-6 has been associated with altered and, in some instances, increased antibody responses to certain viruses and can be reactivated from latency.^43^ Curiously, patients with MIS can show enhanced antibody responses to SARS-CoV-2. Biswas et al.^42^ concluded that additional research may be warranted to determine whether iciHHV-6 is commonly observed in MIS patients and, if so, whether it may play a part in its pathogenesis.

Finally, since the introduction of SARS-CoV-2 vaccines, multiple cases of Pityriasis Rosea-Like Lesions (PR-LE) have been reported.^13^, ^44^ Català et al.^44^ noted PR-LE in 4.9% of a cohort of 405 patients, as well as VZV and HSV reactivation in 10.1% and 3.7%, respectively. PR-LE following the administration of other vaccines had been previously described. Some authors distinguish vaccine-induced PR, possibly due to reactivation of HHV-6 and/or -7, from vaccine-induced PR-LE, which may occur as a delayed hypersensitivity response to a vaccine, in the absence of any signs of HHV-6/7 reactivation.^45^ Differentiation between “true” PR and a PR-like eruption is difficult and virological studies of HHV-6/7 reactivation are performed only in a minority of cases.^45^ HHV-6 status has not been investigated in most cases of SARS-CoV-2 vaccine-induced PR-like eruption cases reported to date, except in 2 cases related to the Pfizer BioNTech (BNT162b2) vaccine in which high HHV‐6 antibody titers were detected.^46^

Given concerns over an increase in VZV and HSV reactivation after vaccination with Tozinameran, Brosh-Nissimov et al.^32^ studied oropharyngeal shedding of herpesviruses, including HHV-6, before and after vaccination. They reported no significant differences in positivity for any herpesvirus between pre-vaccination and post-vaccination samples. The EUDRAvigilance database (Table 1) records 9654 cases of HHV-6: 1 after CX-024414; 9652 after Tozinameran; and 1 after CHADOX1 NCOV-19.^11^

### Human herpesvirus 8

Kaposi Sarcoma-Associated Herpesvirus (KSHV) is an oncogenic virus of the human γ-herpesvirus subfamily and is the etiologic agent of several human cancers, including Kaposi Sarcoma (KS), primary effusion lymphoma, and multicentric Castleman’s disease. It is also associated with a hyperinflammatory syndrome named Kaposi sarcoma herpesvirus-associated inflammatory cytokine syndrome, which can be fatal in some patients.^47^ The seroprevalence of KSHV infection in the general population of North America is less than 10%, but in most of sub-Saharan Africa, the overall seroprevalence exceeds 50%. In the United States and Europe, HHV-8 antibodies are present in ∼30% of HIV-1-infected men who have sex with men.^48^ KSHV can be diagnosed by various methods, including quantification of viral DNA in blood, HHV8-LANA-1 immunostaining in biopsy specimens, and serological testing.

The KSHV viral life cycle consists of a quiescent latent phase and a replicative lytic phase. While the establishment of latency enables persistent KSHV infection and evasion of the host’s immune system, lytic replication is essential for the dissemination of the virus between hosts and within the host itself. The transition between these phases, known as lytic reactivation, is controlled by a complex set of environmental, host-related, and viral factors. One such trigger is COVID-19 infection, and SARS-CoV-2-encoded proteins can induce lytic reactivation of KSHV through manipulation of intracellular signaling pathways.^49^ This has been demonstrated in clinical practice in a patient that had been recently hospitalized for COVID-19 infection and developed skin lesions consisting of bluish-red maculopapular, a biopsy of which established a diagnosis of KS. Transmission electron microscopy of the skin biopsy revealed the presence of both COVID-19 and HHV-8.^50^ It is proposed that SARS-CoV-2 contributes to the development of a hyper inflammatory state, leading to HHV-8 proliferation and consequent recurrence of Kaposi sarcoma.

The EUDRAvigilance database (Table 1) records 3 cases of Kaposi sarcoma, all after vaccination with Tozinameran.^11^

## Conclusion

**Figure 1:**
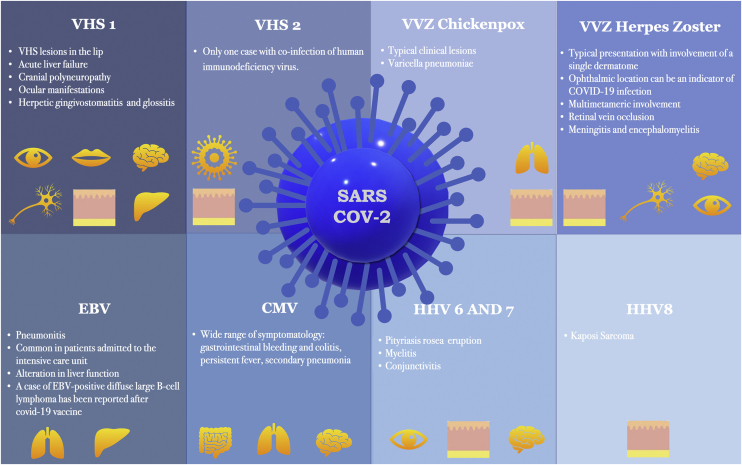
Manifestations of herpesvirus infections associated with COVID-19. VHS-1, Herpes Simplex Virus Type-1; VHS-2, Herpes Simplex Virus Type-2; VVZ, Varicella-Zoster Virus; EBV, Epstein-Barr Virus; CMV, Cytomegalovirus; HHV-6, Herpes Virus-6; HHV-7, Herpes Virus-7; HHV8, Herpes Virus-8.

Numerous articles have linked COVID-19 infection with coinfection or reactivation of human herpes viruses. Furthermore, evidence suggests that human herpesvirus infection may constitute a prognostic marker of COVID-19 infection and even be responsible for many of the manifestations initially attributed to SARS-CoV-2. Recent reports indicate that in addition to the SARS-CoV-2 virus, all COVID-19 vaccines approved to date in Europe are capable of inducing herpesvirus reactivation. Fig. 1 shows the main manifestations of herpesvirus infections associated with COVID-19 (Fig. 1). It is important to take this information into account and to consider all viruses of the Herpesviridae family when managing patients with COVID-19 infection or recently vaccinated against COVID-19.

## Financial support

None declared.

## Authors’ contributions

Alba Navarro-Bielsa: Approval of the final version of the manuscript, critical literature review, data collection, analysis and interpretation, effective participation in research orientation, intellectual participation in propaedeutic and/or therapeutic management of studied cases, manuscript critical review, preparation and writing of the manuscript, statistical analysis, study conception, and planning.

Tamara Gracia-Cazaña: Approval of the final version of the manuscript, critical literature review, data collection, analysis and interpretation, effective participation in research orientation, intellectual participation in propaedeutic and/or therapeutic management of studied cases, manuscript critical review, preparation and writing of the manuscript, statistical analysis, study conception, and planning.

Beatriz Aldea-Manrique: Approval of the final version of the manuscript, critical literature review, data collection, analysis and interpretation, effective participation in research orientation, intellectual participation in propaedeutic and/or therapeutic management of studied cases, manuscript critical review, preparation and writing of the manuscript, statistical analysis, study conception, and planning.

Isabel Abadías-Granado: Approval of the final version of the manuscript, critical literature review, data collection, analysis and interpretation, effective participation in research orientation, intellectual participation in propaedeutic and/or therapeutic management of studied cases, manuscript critical review, preparation and writing of the manuscript, statistical analysis, study conception, and planning.

Adrián Ballano: Approval of the final version of the manuscript, critical literature review, data collection, analysis and interpretation, effective participation in research orientation, intellectual participation in propaedeutic and/or therapeutic management of studied cases, manuscript critical review, preparation and writing of the manuscript, statistical analysis, study conception, and planning.

Isabel Bernad: Approval of the final version of the manuscript, critical literature review, data collection, analysis and interpretation, effective participation in research orientation, intellectual participation in propaedeutic and/or therapeutic management of studied cases, manuscript critical review, preparation and writing of the manuscript, statistical analysis, study conception, and planning.

Yolanda Gilaberte: Approval of the final version of the manuscript, critical literature review, data collection, analysis and interpretation, effective participation in research orientation, intellectual participation in propaedeutic and/or therapeutic management of studied cases, manuscript critical review, preparation and writing of the manuscript, statistical analysis, study conception, and planning.

## Conflicts of interest

None declared.
